# Blood Microsampling to Monitor Metabolic Profiles During Physical Exercise

**DOI:** 10.3389/fmolb.2021.681400

**Published:** 2021-05-27

**Authors:** Cindy Nix, Maryam Hemmati, Gaël Cobraiville, Anne-Catherine Servais, Marianne Fillet

**Affiliations:** Laboratory for the Analysis of Medicines, Department of Pharmacy, CIRM, University of Liège, Liège, Belgium

**Keywords:** amino acids, organic acids, running, hemaPEN^®^, UHPLC-MS/MS, targeted metabolomics

## Abstract

Monitoring approaches and technical improvements are key factors to improve a sportsman’s health, training, and recovery after an injury. In this study, a targeted metabolomics approach using microsampling with hemaPEN^®^ was developed to measure changes in blood concentrations of nine amino acids and four organic acids before, during, and after exercise. The aim of this research project was to investigate if a reliable monitoring of metabolite levels during sports activity can be achieved by collecting one drop of whole blood at different time points. A hemaPEN device is an easy-to-use and noninvasive microsampling technique designed to collect four accurate and precise blood volumes simultaneously (10.96 µl). Twenty healthy volunteers between 19 and 30 years of age were included in this study. Physical activity consisted in running as fast as possible 1,600 m after 400 m warm-up. One drop of blood was collected at five time points: before exercise, after 800-m running, after 1,600 m, and 30 min and 60 min after finishing the exercise. The influence of physical activity on metabolite levels was evaluated using two ultrahigh-performance liquid chromatography coupled to tandem mass spectrometry (UHPLC-MS/MS) methods. Analytical performance criteria such as metabolite stability, method precision, trueness, and accuracy were found to be satisfactory. Expected significant metabolic changes were identified for lactic acid, main TCA cycle intermediates, and some amino acids (e.g., creatinine, choline, and taurine). This preliminary study performed on a small cohort demonstrated a high interest of using microsampling for fluxomics analysis, not only to collect quickly and easily biological samples during sports events but also because it is much easier to store and to process the samples than classical plasma/serum samples obtained by venipuncture. The present results open new avenue for fluxomics analysis in the context of health care.

## Introduction

Exercise is one key factor to sustain good health (Kelly et al., 2020). Indeed, it is widely proven that physical activity reduces the risk of obesity (Mielke et al., 2019), cardiovascular diseases (Porter et al., 2019), diabetes, hypertension (Aune et al., 2015), and depression (Matta et al., 2021). The World Health Organization (WHO) recommends at least 150 min of moderate exercise or 75 min of intense exercise per week for adults. Muscle strengthening activities and reduced sedentary time also constitute two critical points to keep fit. Despite all the recognized benefits of sports, worldwide around 1 in 4 adults is not sufficiently physically active (WHO, 2020). In order to promote physical exercise, various awareness campaigns and programs have recently been set up, such as the Project Smart which promotes physical activity for children through games (Julien et al., 2021) or the global action plan of the WHO to encourage sport activities (WHO, 2018). In order to avoid any risk of injury, it is crucial that sports practice is supervised and adapted to each person. For athletes, this consideration is particularly crucial since an injury can have a big impact on their health and their career as well as financial repercussions. Currently, the number of competitions in which athletes participate is increasing. Therefore, athletes intensify their training to be always competitive. Nevertheless, health professionals agree that overloading training and competitions can have serious consequences for the health of athletes (Soligard et al., 2016). To avoid the risk of injury, several strategies are available.

In those contexts of amateur and professional sports, fluxomics could be an interesting approach to implement personalized athlete monitoring to reduce the risk of injury, adapt the training, and speed up recovery after injury if any (Al-Khelaifi et al., 2019). Indeed, the measurement of metabolite levels in individuals at different time frames provides information concerning the evolution of their physiopathological state. For example, several studies have shown that exercise induced metabolic changes. Lewis *et al.* demonstrated that the concentrations of niacinamide, glucose-6-phosphate, pantothenate, and succinate increased in plasma after an exercise (Lewis et al., 2010). More recently, Stander *et al.* have shown that the serum concentrations of carbohydrates, fatty acids, TCA cycle intermediates, and ketones were increased after a marathon, while the levels of amino acids were reduced (Stander et al., 2018). Prado et al. even proposed the term “sportomics” to qualify the use of “-omics” sciences to better understand the metabolic changes induced by a physical activity (Prado et al., 2017). They performed untargeted metabolomics in urine before and after a soccer match. Different categories of metabolites were found to be interesting, among which are organic acids (Prado et al., 2017).

**FIGURE 1:**
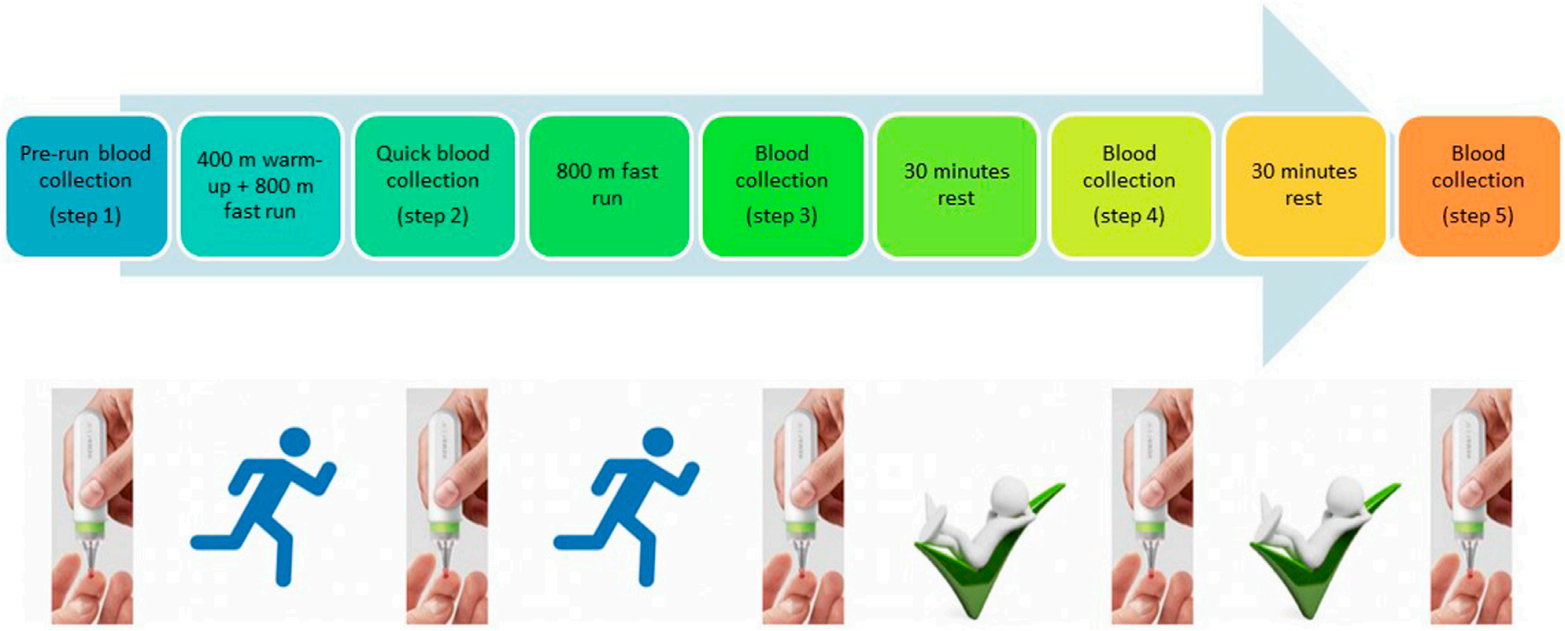
Design of the study.

Although several studies showing metabolomic changes during or after physical activity have already been published (Sakaguchi et al., 2019), none allowed athletes to collect blood by themselves directly at the training site. In this study, we aimed to make the proof of concept that well-known biomarkers such as lactic acid or creatinine can easily be monitored during physical activity using a noninvasive and easy-to-handle microsampling device. Blood collection was achieved using a microsampling device named hemaPEN^®^ (Trajan Scientific and Medical, VIC, Australia). This pen-shaped device allows the accurate and precise collection of samples from a single drop of blood and, depending on the studied compounds, is influenced in a limited way or not influenced by hematocrit compared to classical DBS (Deprez et al., 2019; Protti et al., 2020). Blood is absorbed by four capillaries and transferred to four paper discs. Each capillary collects 2.74 µl of blood. This device can be used everywhere by everyone without specific training (Trajan-HemaPEN brochure, 2020) (cf. Figure 1). In this study, we investigated whether it is possible to monitor metabolism changes in a reliable manner from 2.74 µl of blood not only before and after the exercise but also during the effort.

## Materials and Methods

### Chemicals and Reagents

L-asparagine (≥98%), choline chloride (≥99%), creatine (≥99.5%), creatinine (≥98%), L-leucine (≥98%), L-methionine (≥98%), L-proline (≥99.5%), taurine (≥99%), L-valine (≥98%), 2-hydroxybutanoic acid, L-lactic acid (≥98%), malic acid (≥99%), and 2-oxo-glutaric acid (≥99%) were purchased from Sigma Aldrich (St. Louis, MO, United States). L-lactic acid-d_3_ and choline-d_9_ were obtained from Toronto Research Chemicals (Toronto, ON, Canada). L-asparagine ^13^C_4_ (≥99%) and L-leucine-d_3_ (≥99%) were obtained from Euriso-top (Saint-Aubin, France). Acetonitrile, formic acid, trifluoroacetic acid, isopropanol, methanol, and water (MS-grade) were bought from Biosolve (Valkenswaard, The Netherlands). hemaPEN^®^ devices were obtained from Trajan Scientific and Medical (VIC, Australia) and Ostro^®^ 96-well sample preparation plates from Waters Corporation (Dublin, Ireland). MiniCollect^®^ safety lancets were purchased from Greiner Bio-One (Vilvoorde, Belgium).

### Healthy Volunteers

The protocol of this study was reviewed and approved by the Ethical Committee of the University of Liège (Liège, Belgium), Nr Eudra CT: B7072020000041; ref: 2020/182. The participants provided their written informed consent to participate in the study. Twelve women and eight men between 19 and 30 years of age with a body mass index (BMI) between 18.5 and 25 were included in this study. The participants did not suffer from any chronic respiratory, inflammatory, cardiovascular, or metabolic diseases and were not smokers. The participants did not have any fever, and they have not been infected with COVID-19. They exercised at least 1 h per week (4.2 h/week in average). To minimize the impact of nutrition on metabolic changes, they had standard meals (breakfast and lunch) on the day of running and no excess food or drink the day before the exercise. The parameters of all the participants are described in Supplementary Table S1.

### Physical Exercise

The physical exercise consisted of running 400 m as a warm-up that was followed by running 1,600 m at high speed on athletic tracks.

### Sample Collection

Blood samples were collected at five different time points. At every time point, four replicate samples were collected. The first sample was collected at rest, shortly before running (Step 1), the second sample after running of 400-m warm-up and 800-m running (Step 2), the third sample after the completion of 1,600-m running (Step 3), and the fourth and fifth samples at 30 min (Step 4) and 60 min (Step 5) after finishing the exercise, respectively. Blood was collected with a hemaPEN^®^ device *via* a finger prick with a safety lancet (Figure 1).

### Sample Storage

After collection, the hemaPEN^®^ devices were kept at ambient temperature for 2 h to allow the samples to dry. The devices were then stored at −20°C before analysis.

### Stock Solutions of Amino Acids and Organic Acids

Aqueous stock solutions of nine amino acids and four organic acids were prepared at specific concentrations: 200 mM choline chloride, 100 mM L-methionine, 150 mM L-asparagine, 150 mM L-leucine, 100 mM creatine, 200 mM taurine, 500 mM L-proline, 300 mM L-valine, 500 mM creatinine, 800 mM L-lactic acid, 150 mM 2-hydroxybutanoic acid, 120 mM malic acid, and 80 mM 2-oxoglutaric acid. Stocks solutions were stored at −80°C. Aqueous stock solutions of 10 mM L-lactic acid-d_3_, choline-d_9_, L-asparagine ^13^C_4_, and L-leucine-d_3_ were prepared to be used as internal standards. These solutions were stored at −80°C.

### Calibration Standards and Quality Control

Calibration solutions were prepared by diluting aqueous stock solutions in human blood to reach the targeted concentrations. Separate calibration curves were prepared for the analytes in the range of their endogenous concentrations. Calibration ranges were as follows: 15–150 µM for 2-hydroxybutanoic acid, 640–6400 µM for lactic acid, 6–60 µM for malic acid, 2.4–24 µM for 2-oxoglutaric acid, 14–140 µM for asparagine, 5.5–55 µM for choline, 92.5–925 µM for creatine, 27–270 µM for creatinine, 28–280 µM for leucine, 5.5–55 µM for methionine, 55–550 µM for proline, 41.5–415 µM for taurine, and 56–560 µM for valine.

Five calibration levels were used to construct calibration curves. Concentration at the upper limit of the calibration curves (C5) was considered as the 100% concentration. This C5 solution was diluted to obtain the other levels: 10%=C1 (lowest level of the calibrations curves), 25%=C2, 50%=C3, and 75%=C4. The calibration standards were prepared with hemaPEN^®^ devices and extracted according to the sample preparation procedure, and each calibration standard was injected six times. A quality control (QC) sample was prepared at C3 and injected after every twenty injections to check the system performance during the batch analysis.

### Sample Preparation

Using Trajan’s specially designed tool, the hemaPEN^®^ devices were opened and the cartridge containing the four replicate samples removed. One paper disc was used for this study, and the other three were kept in the cartridge in a sealed box at −20°C for possible future analyses.

The extraction solvent was a mixture of acetonitrile and water (60:40, v/v) containing the internal standards in the concentrations as follows: 80 μM for L-lactic acid-d3, 1.25 μM for choline-d9, 5 μM for L-asparagine 13C4, and 7.5 μM for L-leucine-d3. The hemaPEN^®^ paper disks were placed in an Ostro™ 96-well plate (Waters Corporation, Dublin, Ireland) and were incubated with 200 µl extraction solvent for 5 min without agitation followed by an agitation step of 5 min using a ThermoMixer C (Eppendorf, Aarshot, Belgium) at 20°C and 850 rpm. The samples were then collected in a 96-well plate (Agilent Technologies, Waldbronn, Germany) by passing them through the Ostro™-plate using a vacuum manifold.

Before the analysis of organic acids by UHPLC-MS/MS, a volume of 100 µl of the extracted samples was evaporated during 60 min at 40°C in a CentriVAP Concentrator (LabConco, Kansas-City, MO, United States). The dried residues were reconstituted in 50 µl water and analyzed by UHPLC-MS/MS. The sample preparation procedure is described in Supplementary Figure S1.

### UHPLC-MS/MS Analysis

The samples containing the amino acids and organic acids were analyzed with an ultrahigh-performance liquid chromatography–tandem mass spectrometry (UHPLC-MS/MS) method that was published previously by our group (Kok et al., 2019). LC-MS/MS analyzes were conducted on an Agilent^®^ 1290 Infinity system coupled to an Agilent^®^ 6495 triple quadrupole mass spectrometer.

### Sample Randomization

Samples were analyzed in a randomized way to ensure that the results obtained are not influenced by the order of analysis.

### Stability Evaluation

The stability of the analytes in the autosampler was assessed. To evaluate the stability in the autosampler, three samples at concentration level C1 (first level of the calibration curves) and three samples at concentration level C5 (upper limit of the calibration curve) were analyzed immediately after preparation and after 24-h storage at 4°C in the autosampler. The responses obtained after 24 h were compared to the responses obtained immediately after sample preparation.

Long-term stability was assessed using one of the four replicate samples collected with the hemaPEN^®^ device. One paper sample disc collected after 1,600 m of running was analyzed after 5 months of storage and compared to the freshly analyzed samples. This study was possible because four replicates per sample are collected simultaneously with one hemaPEN^®^ device. Stability is considered satisfactory if the responses do not vary more than 15% compared to fresh samples (Smith, 2012).

### Performance Criteria of Analytical Methods

#### Response Function, Trueness, Precision, and Accuracy

For all the studied compounds, the most appropriate regression model as well as the trueness, the precision, and the accuracy of the methods were obtained performing the prevalidation of the methods. Calibration curves with five levels were prepared independently on three different days. The most suitable regression models were chosen according to the obtained accuracy profiles. The acceptance limits and the maximum risk of having future measurements falling out of these acceptance limits were set at ±20%. All accuracy profiles are presented in Supplementary Figure S2. Trueness of the analytical method was evaluated using the relative bias (%) at five concentration levels. Within-run precision was assessed by the repeatability (RSD%) and between-run precision by the intermediate precision (RSD%).

#### Matrix Effect

The matrix effect was assessed according to the method described by Matuszewski et al. (2003) at two concentration levels (midrange of the calibration curve and the upper limit of the calibration curve). For amino acids, neat standard solutions containing the appropriate concentration of all the amino acids were prepared in acetonitrile/water (6:4). For organic acids, a neat standard solution containing the appropriate concentration of all the organic acids was prepared in water. Post-extraction spiked matrices were prepared by extracting blank blood. For amino acids, extracted blank blood was directly spiked with a mixture containing all the studied amino acids at the appropriate concentration. For organic acids, 100 µl of extracted blank blood was evaporated during 60 min at 40°C in a CentriVAP Concentrator (LabConco, Kansas City, MO, United States) and reconstituted with 50 µl of water containing the appropriate concentration of all the organic acids studied. The matrix effect was calculated by dividing the peak areas obtained with the post-extraction spiked matrices by the peak areas obtained with the neat standard solutions. Neat standard solutions and post-extraction spiked matrices were prepared in triplicates for each concentration level.

#### Carryover

Carryover was assessed according to the guideline on bioanalytical method validation from the European Medicines Agency (EMA) (Smith, 2012). A solution at the highest concentration level was injected three times. After each injection, a blank was injected. For all compounds, the peak area obtained in blanks should not exceed 20% of the peak area obtained at the lowest level of the calibration curves. For the internal standards, the peak area obtained should not exceed 5%. Carryover was calculated according to the formula below:

$$Carryover\;\left(\%\right)=\;\frac{Peak\;area\;obtained\;in\;a\;blank\;after\;an\;injection\;of\;a\;solution\;at\;the\;highest\;concentration\;level}{Peak\;area\;obtained\;at\;the\;lowest\;level\;of\;the\;calibration\;curve}.$$

### Data Analysis

Data acquisition was performed with MassHunter Data Acquisition software (B.08.02, Agilent Technologies, Waldbronn, Germany). Data analysis was performed with Quantitative Analysis software (version 10.1, Agilent Technologies, Waldbronn, Germany). Peak areas corresponding to the endogenous concentrations of studied compounds in blood used to prepare calibration curves were subtracted from the values obtained for the calibration solutions (Vishwanathan et al., 2000). The concentration obtained at each step of the study was divided by the concentration obtained at step 1 (at rest before exercise) to obtain a change of magnitude factors. The means of the change of magnitude factors obtained at each step were compared with a one-way ANOVA test for data with a normal distribution and a Friedman test for data not satisfying the normality test. These statistical analyzes were performed using GraphPad prism 6 and as well as the construction of the different graphs. Results of prevalidation were computed with e-noval 4.1 software (Pharmalex, Mont-Saint-Guibert, Belgium).

## Results

In this study, two previously developed UHPLC-MS/MS methods were used to quantify nine amino acids and four organic acids in whole blood after microsampling. Five samples were collected by each participant before, during, and after a physical activity consisting in 1,600 m of intensive running. This study focused on 13 metabolites because the influence of physical activity on their blood concentration is well-described in the literature (Stander et al., 2018; Schranner et al., 2020). Indeed, the aim of our study was not to discover new biomarkers but to investigate the potential of microsampling using hemaPEN^®^ devices for fluxomics analysis.

### Analytical Performance of the Methods

For all the studied compounds, the most adequate response functions to quantify the analytes over the entire range of concentrations were determined by applying different regression models and selecting the most suitable accuracy profiles. The selected models for which the tolerance limits are included within the acceptance limits (±20%) as well as the accuracy profiles are presented in Supplementary Figure S2.

In term of trueness, relative bias was below ±8.63% for all the organic acids and below ±8.24% for all the amino acids at the five investigated concentration levels. For within- and between-run precision, repeatability (RSD%) and intermediate precision (RSD%) were below 13.9% for all the compounds at the five concentration levels. The results obtained for trueness, repeatability, and intermediate precision are presented in Supplementary Table S2.

#### Stability

For all the studied compounds, the responses obtained directly after preparation and after 24-h storage in the autosampler or after 5 months storage at −20°C do not vary by more than 15%, except for 2-oxoglutaric acid, methionine, creatine, and taurine at long-term storage. For 2-oxoglutaric acid and methionine, concentrations obtained after 5-month storage were 35.1 and 56.5% lower than the nominal concentrations, respectively. Concentrations of creatine and taurine obtained after 5-month storage at −20°C were 17.6 and 15.7% higher than the nominal concentrations, respectively. Results concerning the stability in the autosampler and the long-term stability are presented in Supplementary Tables S3, S4, respectively**.**

#### Matrix Effect and Carryover

The matrix effect represents the influence of the matrix on the ionization of the compounds of interest in the ESI source (Matuszewski et al., 2003). Indeed, human blood contains a large number of compounds. These compounds can interfere with the ionization of the quantified analytes. A matrix effect lower than 100% means that the matrix reduces ionization efficiency, while a matrix effect higher than 100% means that the signal is enhanced in the matrix. The obtained results are presented in Supplementary Table S5.

The matrix effect was lower than 100% (in average 76.6%) for all the compounds, except for asparagine (117%). The ionization of creatine does not seem to be influenced by the matrix (99.9%). For creatinine, proline, valine, and methionine, the matrix has a more important impact on the ionization process. The results obtained at the different concentration levels were comparable (Supplementary Table S5).

Concerning the carryover, the peak areas obtained in the blank samples did not exceed 2.07% of the peak area obtained at the lowest level of the calibration curve, for all the compounds. Percentages of carryover are presented in Supplementary Figure S3.

### QC Samples

The relative standard deviation (RSD) of the concentrations obtained for the QC samples was below 10.8% for all the amino acids and below 10.0% for all the organic acids. In clinical laboratories, the Levey–Jennings charts are commonly used to report values obtained for QC samples. An example of the Levey–Jennings chart obtained with the QC samples injected along all the analyses of runners’ samples are given in Supplementary Figure S4. This type of graph is usually interpreted, thanks to the Westgard rules. According to these rules, no QC sample should exceed ±3 SD; four consecutive QC samples should not exceed +1 SD or −1 SD; two consecutive QC samples should not exceed ±2 SD, and ten consecutive QC samples should not fall on one side of the mean (Dasgupta and Wahed, 2014). In this case, QC samples met all these criteria.

### Monitoring of Metabolites Levels Before, During, and After Exercise

**FIGURE 2:**
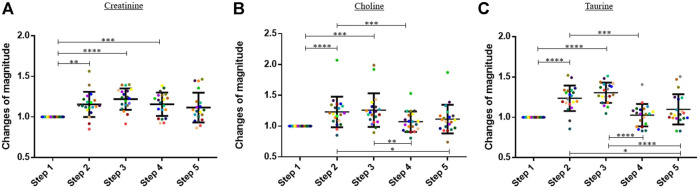
Graphs representing the “changes of magnitude” obtained during the five steps of the study. Step 1: before physical exercise, step 2: after 400-m warm-up and 800-m running, step 3: after the completion of 1,600-m running, step 4: 30 min after finishing the exercise, and step 5: 60 min after finishing the exercise. Each color represents one participant. Black lines represent the mean ± SD of the change of magnitude factor obtained at each step (*n* = 20). **(A)** Creatinine, **(B)** choline, **(C)** taurine: amino acids for which an increase in blood concentration was observed during the exercise (*, *p*-value < 0.05; **, *p*-value < 0.01; ***, *p*-value < 0.001; ****, and *p*-value < 0.0001).

In this study, the blood concentrations of three amino acids, namely, creatinine, choline, and taurine, increased significantly during the exercise (Figure 2). Results are expressed in change of magnitude factors compared to the blood concentration before running. The blood concentration of creatinine increased by a factor 1.22 during the exercise, and no statistically significant decrease was observed during the 60 min following the physical activity. The blood concentration of choline and taurine increased by factors 1.21 and 1.31 during running, respectively. A decrease was already observed 30 min after the end of the physical activity. The means of the change of magnitude factors observed at each step were compared with a one-way ANOVA test for creatinine and taurine and with a Friedman test for choline.

**FIGURE 3:**
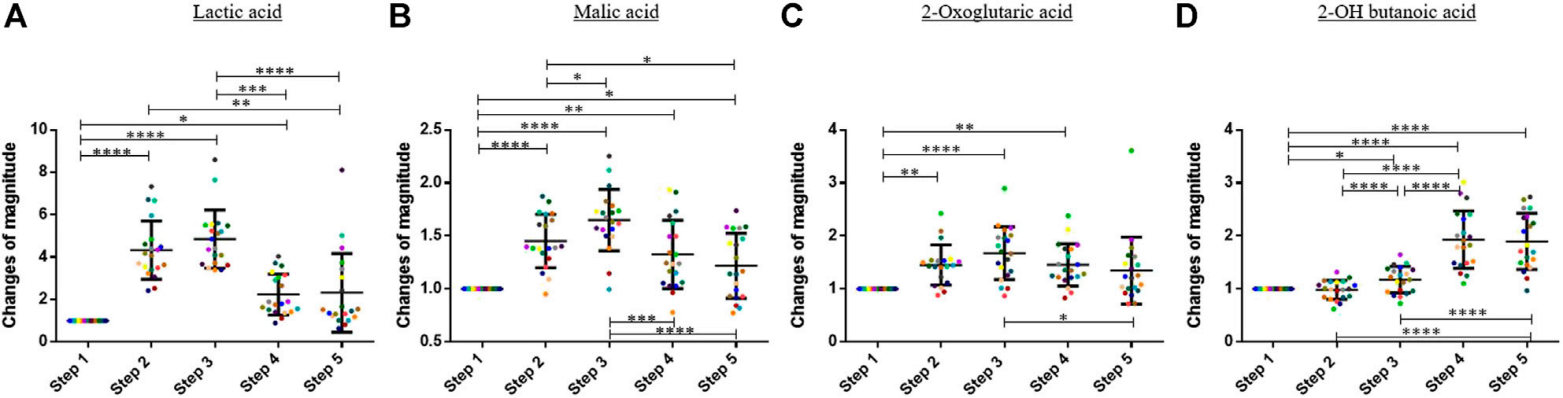
Graphs representing the “changes of magnitude” obtained during the five steps of the study. Step 1: before physical exercise, step 2: after 400-m warm-up and 800-m running, step 3: after the completion of 1,600-m running, step 4: 30 min after finishing the exercise, and step 5: 60 min after finishing the exercise. Each color represents one participant. Black lines represent the mean ± SD of the change of magnitude factor obtained at each step (*n* = 20). **(A)** Lactic acid, **(B)** malic acid, **(C)** oxoglutaric acid, **(D)** 2-OH butanoic acid: organic acids for which an increase in blood concentration was observed during and/or after the exercise (*, *p*-value < 0.05; **, *p*-value < 0.01; ***, *p*-value < 0.001; ****, and *p*-value < 0.0001).

Regarding the organic acids, the 1,600-m running induced an increase of the blood concentration of lactic acid, malic acid, and 2-oxoglutaric acid. The blood concentration of these compounds decreased during the 60 min after finishing the exercise (Figure 3). On average, the blood concentration of lactic acid, malic acid, and 2-oxoglutaric acid increased by factors 4.86, 1.65, and 1.67 between step 1 and step 3, respectively. A Friedman test was performed to compare the means of the change of magnitude factors for lactic acid, malic acid, and 2-oxoglutaric acid.

For 2-OH butanoic acid, the highest blood concentration was observed 30 min after finishing the exercise (*see* Figure 3). A slight decrease was observed at step 5, but this decrease was not statistically significant. The results obtained at the different steps were compared with a one-way ANOVA for this compound.

**FIGURE 4:**
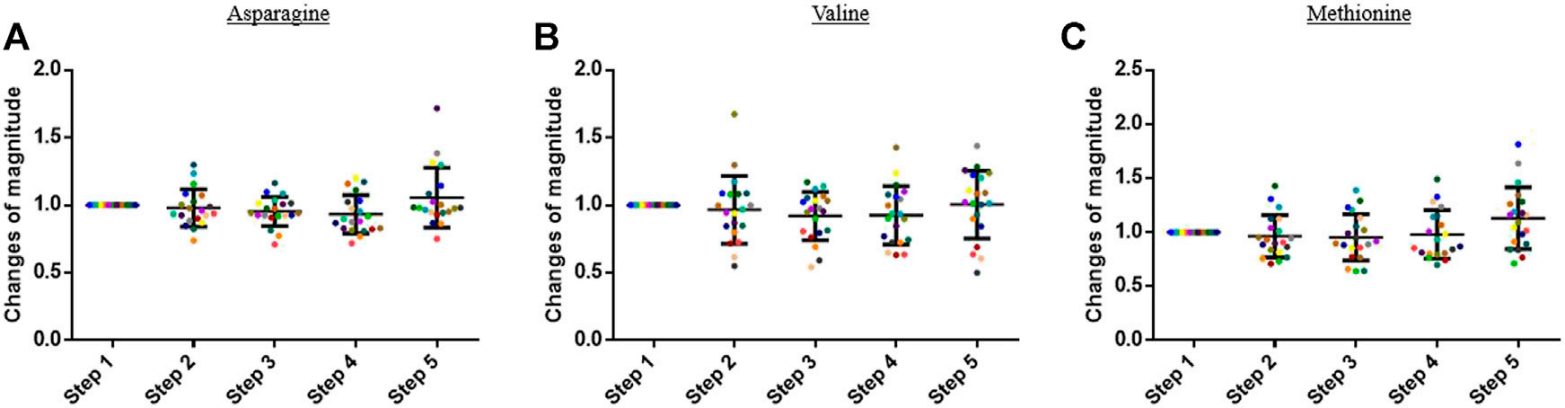
Graphs representing the “changes of magnitude” obtained during the five steps of the study. Step 1: before physical exercise, step 2: after 400-m warm-up and 800-m running, step 3: after the completion of 1,600-m running, step 4: 30 min after finishing the exercise, and step 5: 60 min after finishing the exercise. Each color represents one participant. Black lines represent the mean ± SD of the change of magnitude factor obtained at each step (*n* = 20). **(A)** Asparagine, **(B)** valine, and **(C)** methionine: examples of compound for which no change in blood concentration was observed during the study.

The concentration of the other analyzed compounds did not significantly vary in this study. Some examples are presented in Figure 4.

## Discussion

In this study, we have demonstrated the potential of microsampling to follow up easily several metabolites. The hemaPEN^®^ device offers many advantages compared to classical blood sampling. Indeed, this type of blood collection can be done by everyone and everywhere. The volume of blood collected is low, and thus, multiple collections is not an issue. Moreover, this type of sampling is less invasive than a venipuncture. The integrity of the sample is guaranteed because the paper discs on which the blood is absorbed are not accessible once the sample has been collected. They are protected from light and from humidity, thanks to a fully integrated desiccant. Compared to other microsampling devices that are available, for example, in the VAMS device from Neoteryx or the new Microsampling Wing™ from Shimadzu, four replicates of the same sample are collected with one drop of blood. This constitutes a key advantage because one paper disc can be analyzed, while the three others can be stored for future analyzes or used as backup if an issue is encountered during the first analysis. In this study, we demonstrated the potential of the hemaPEN^®^ for the follow-up of athletes, but many other applications could be developed in the near future such as drug therapeutic monitoring or anti-doping tests. The World Anti-Doping Agency (WADA) recommends long-term sample storage and re-analysis programs for a more effective detection of doping (Word Anti-Doping Agency, 2020). In the near future, WADA also wants to develop guidelines for the collection, transport, analysis, and storage of DBS samples to implement this type of sampling in routine analyses. In this context, the hemaPEN^®^ device could be interesting for anti-doping tests (World Anti-Doping Agency, 2020).

In this study, the most appropriate regression models were chosen, thanks to the accuracy profiles with acceptance limits below ±20%. Both UHPLC-MS/MS methods showed acceptable trueness and precision. The stability of the studied analytes in the autosampler conformed to EMA guidelines. The long-term stability at −20°C was satisfactory, except for 2-oxoglutaric acid, methionine, creatine, and taurine. Depending on the metabolites, stability might be affected during storage. Nevertheless, since relative quantification is performed in this kind of monitoring, the impact of the degradation should not be an issue, provided that all the compared samples are prepared simultaneously.

Importantly, we observed that the Levey–Jennings charts obtained for the QC samples during the analysis of the runners’ samples met all the criteria described in the Westgard rules. All the samples were therefore analyzed in appropriate conditions.

Since the cohort was small (20 individuals), statistical analysis was performed using the one-way ANOVA or Friedman test. Since the reliability of the analytical process involving self-microsampling was demonstrated in this study, it would be interesting to conduct additional studies involving larger cohorts to investigate the influence of different parameters such as BMI, sex, age, ethnicity, intensity of the efforts, and genetic predisposition, on metabolic changes. Such studies could include a broader panel of targeted metabolites or could be done by untargeted metabolomics. In those cases, dedicated multivariate analysis should be employed (i.e., PCA analysis).

Concerning the change in magnitude factors observed in our study, low variation in blood concentrations of amino acids was observed. Nevertheless, an increase in the blood concentration of taurine and creatinine was observed. Cuisinier *et al.* and Medelli *et al.* already described an increased concentration of taurine in plasma after a marathon (Cuisinier et al., 2001) and after a cycling competition (Medelli et al., 2003). Several other studies mentioned an increase in serum creatinine levels after physical activity (Hodgson et al., 2017). This increase in creatinine can be explained by an increased muscle breakdown (Hodgson et al., 2017; Omassoli et al., 2019). Pechlivanis *et al.* observed a decrease in leucine and valine serum concentrations after sprint running (Pechlivanis et al., 2013). After a marathon, Stander *et al.* observed a reduced concentration of methionine, valine, and leucine (Stander et al., 2018). Nevertheless, for the amino acids, conclusions are not consistent across all the studies (Schranner et al., 2020). The duration and the intensity of exercise or the fitness of the participants could explain these discrepancies between studies. A decrease in blood concentrations of amino acids can be explained by the fact that the body activates the catabolism of these molecules to find energy when carbohydrate stores are depleted. In our study, exercise duration was short. This is probably why the body may not have used all of its carbohydrate stores. Therefore, the concentrations of the amino acids were not decreased. Future studies with a longer exercise duration should be conducted to confirm this hypothesis.

Concerning the organic acids, lactate represents one key compound. Indeed, several articles already described an increase in lactate during exercise (Pechlivanis et al., 2013; Gáspari et al., 2015). This accumulation in lactate reflects the shift from an aerobic to an anaerobic energy production system. Monitoring this shift constitutes an essential parameter to adapt athletes’ training in endurance sports (Etxegarai et al., 2019). In our study, the blood concentration of lactic acid was found to increase significantly during exercise, showing the potential of our method to follow up athletes. Thirty minutes after the end of the physical exercise, the concentration of lactic acid decreased to reach a concentration near the nominal one. The blood concentration of two other organic acids, that is, malic acid and 2-oxoglutaric acid, intermediates in the TCA cycle, also increased during physical effort. This finding corresponds to the conclusions previously obtained in other studies. Indeed, Stander *et al.* found elevated serum concentrations of these molecules after a marathon (Stander et al., 2018). Recently Schranner *et al.* published a review bringing together 27 studies and 57 experiments. They concluded that the blood concentrations of intermediates of the TCA cycle such as malate or 2-oxoglutaric acid were upregulated by exercise as well as the concentration of lactate. This increase in lactate was particularly noted with high-intensity exercise. The concentration of these three organic acids decreased during the 60 min after physical activity, as observed in our study (Schranner et al., 2020). Schranner’s review also reported an increase in the concentration of ketone bodies during exercise. In our study, 2-hydroxybutanoic acid, a ketone body, was found to increase during and after exercise. Therefore, the conclusions obtained in this study for the organic acids are in agreement with the literature.

Finally, this study demonstrates that microsampling with hemaPEN^®^ followed by targeted metabolomics has a great potential for the follow-up of metabolic profiles. This study used two UHPLC-MS/MS methods to quantify 13 metabolites using only 2.74 µl of whole blood and offers the main advantage that sampling can be performed directly at the training site without special skill. In the future, targeted and untargeted metabolomics approaches on broader cohorts could be investigated to expand the panel of applications.

## Data Availability

The raw data supporting the conclusions of this article will be made available by the authors, without undue reservation.

## Abbreviations

EMA, European Medicines Agency; ESI, electrospray ionization; MS/MS, tandem mass spectrometry; MRM, multiple reaction monitoring; NMT, neuromuscular training; QC, quality control; RSD, relative standard deviation; SD, standard deviation; TCA, tricarboxylic acid; UHPLC, ultrahigh-performance liquid chromatography; VAMS, volumetric absorptive microsampling; WADA, World Anti-doping Agency.
