# Supplementary material for: Blood Microsampling to Monitor Metabolic Profiles During Physical Exercise
Source: Front Mol Biosci. 2021 May 27;8:681400. doi: 10.3389/fmolb.2021.681400 (PMC8191458; doi:10.3389/fmolb.2021.681400)
Supplement: Supplementary file 1 [file DataSheet1.docx]

**Supplementary Data Table 1**. Main characteristics of the subjects. CP=contraceptive pill

| Participant | Gender | Age (years) | Height (cm) | Weight (kg) | Specific dietary habits | Medication | Number of hours of sports activity per week |
| --- | --- | --- | --- | --- | --- | --- | --- |
| 1 | F | 25 | 171 | 62 | / | levothyroxine | 1 |
| 2 | M | 22 | 170 | 75 | / | rupatadine | 2 |
| 3 | F | 19 | 172 | 59 | / | CP | 3 |
| 4 | F | 27 | 170 | 70 | / | CP  vitamin D | 1 |
| 5 | M | 27 | 182 | 65 | vegetarianism | / | 1 |
| 6 | M | 27 | 188 | 77 | / | / | 4 |
| 7 | F | 27 | 170 | 54 | vegetarianism | Cetirizine levothyroxine CP | 3 |
| 8 | F | 23 | 156 | 53 | / | / | 2 |
| 9 | M | 20 | 168 | 61 | / | / | 11 |
| 10 | F | 21 | 163 | 50 | / | CP | 8 |
| 11 | M | 29 | 175 | 78 | / | / | 10 |
| 12 | F | 26 | 158 | 54 | / | / | 1 |
| 13 | F | 24 | 166 | 58 | / | CP | 4 |
| 14 | M | 26 | 185 | 70 | / | / | 2 |
| 15 | F | 23 | 172 | 59 | / | CP | 6 |
| 16 | M | 24 | 188 | 69 | / | Cortisone nasal spray | 10 |
| 17 | F | 22 | 164 | 64 | / | CP | 1 |
| 18 | M | 20 | 178 | 65 | / | / | 4 |
| 19 | F | 27 | 162 | 53 | / | CP | 3 |
| 20 | F | 19 | 170 | 57 | / | CP | 7 |

**Supplementary Data Table 2**. Trueness (relative bias %), repeatability (RSD %) and intermediate precision (RSD %) of the analytical method.

| Compounds | Concentration | Trueness  (Relative bias %) | Precision | |
| --- | --- | --- | --- | --- |
|  |  |  | **Repeatability (RSD %)** | **Intermediate precision (RSD %)** |
| ORGANIC ACIDS | | | | |
| 2-Hydroxybutanoic acid | C1  C2  C3  C4  C5 | 0.661  3.41  -3.45  -3.71  2.42 | 4.46  2.52  2.44  10.9  2.84 | 4.64  4.11  3.33  10.9  2.84 |
| Lactic acid | C1  C2  C3  C4  C5 | -1.72  2.87  0.215  -3.02  1.91 | 7.24  9.07  4.11  2.56  4.49 | 7.24  9.07  4.38  6.23  5.18 |
| Malic acid | C1  C2  C3  C4  C5 | -0.312  2.31  -3.58  -2.23  -0.248 | 12.2  4.92  2.87  10.1  8.07 | 12.2  7.33  7.64  11.8  8.68 |
| 2-Oxoglutaric acid | C1  C2  C3  C4  C5 | 0.713  1.15  -8.63  2.38  1.52 | 9.67  11.1  7.72  4.67  3.98 | 9.67  11.1  7.72  6.31  4.64 |
|  | | | | |
| AMINO ACIDS | | | | |
| Asparagine | C1  C2  C3  C4  C5 | 0.372  -2.24  6.11  -6.31  2.78 | 13.9  7.04  5.72  5.20  6.19 | 13.9  8.45  6.75  5.21  6.19 |
| Choline | C1  C2  C3  C4  C5 | -1.71  1.59  4.71  -8.24  4.32 | 6.65  4.95  3.41  2.44  3.41 | 6.85  4.95  3.41  2.44  3.41 |
| Creatine | C1  C2  C3  C4  C5 | 0.408  -1.95  4.97  5.11  2.83 | 12.6  3.31  5.86  4.17  6.26 | 12.6  8.74  6.46  4.29  6.26 |
| Creatinine | C1  C2  C3  C4  C5 | -0.121  -0.553  2.78  -3.11  1.22 | 8.12  3.58  2.48  3.08  3.31 | 8.40  8.32  4.10  4.03  4.01 |
| Leucine | C1  C2  C3  C4  C5 | 0.549  -3.25  7.25  -6.58  3.20 | 8.19  4.64  3.83  4.21  5.60 | 8.19  7.49  7.14  6.32  8.76 |
| Methionine | C1  C2  C3  C4  C5 | 2.51  -4.94  3.16  -0.335  -0.289 | 8.21  6.20  4.71  4.40  3.14 | 8.21  9.07  7.44  4.83  3.14 |
| Proline | C1  C2  C3  C4  C5 | 0.472  -1.43  2.02  -1.39  0.360 | 5.26  5.39  3.28  3.48  1.71 | 6.09  8.55  4.42  3.48  1.94 |
| Taurine | C1  C2  C3  C4  C5 | 1.31  -2.54  2.83  -1.78  0.483 | 5.82  5.63  4.64  3.13  1.43 | 8.63  7.51  4.64  3.13  1.43 |
| Valine | C1  C2  C3  C4  C5 | -0.165  -0.196  1.50  -1.74  0.664 | 9.32  9.90  8.58  7.47  5.10 | 9.32  10.55  8.63  7.47  5.10 |

**Supplementary Data Table 3.** Stability of the samples during 24 hours in the autosampler. Stability assessed at two concentrations (C1=lower limit of the calibration range and C5=upper limit of the calibration range). Results are presented as mean±SD (n=3).

| Compounds | Stability ± SD (%) at concentration level C1 | Stability ± SD (%) at concentration level C5 |
| --- | --- | --- |
| Stability in the autosampler |  |  |
| ORGANIC ACIDS |  |  |
| 2-Hydroxybutanoic acid | 101.0±0.9 | 98.0±0.4 |
| Lactic acid | 100.4±1.0 | 96.8±1.4 |
| Malic acid | 105.5±2.0 | 100.2±2.3 |
| 2-Oxoglutaric acid | 100.3±1.4 | 95.9±2.7 |
|  |  |  |
| AMINO ACIDS |  |  |
| Asparagine | 97.3±5.9 | 102.5±4.0 |
| Choline | 97.1±2.7 | 94.7±3.0 |
| Creatine | 94.4±4.7 | 94.0±1.5 |
| Creatinine | 98.4±1.6 | 96.3±6.9 |
| Leucine | 96.6±6.9 | 99.7±1.8 |
| Methionine | 90.9±3.0 | 93.7±0.9 |
| Proline | 97.1±2.0 | 95.1±3.3 |
| Taurine | 98.9±2.1 | 97.5±2.4 |
| Valine | 107.9±7.0 | 102.4±2.7 |

**Supplementary Data Table 4.** Stability of the analytes in blood samples stored during 5 months at -20°C. Results are presented as mean±SD (n=20).

| Compounds | Stability ± SD (%) | Compounds | Stability ± SD (%) |
| --- | --- | --- | --- |
| ORGANIC ACIDS |  | **AMINO ACIDS** |  |
| 2-Hydroxybutanoic acid | 102.9±11.0 | Asparagine | 94.7±7.4 |
| Lactic acid | 101.9±19.3 | Choline | 102.4±16.3 |
| Malic acid | 96.3±17.6 | Creatine | 117.6±14.9 |
| 2-Oxoglutaric acid | 64.9±13.3 | Creatinine | 101.0±11.2 |
|  |  | Leucine | 87.5±12.1 |
|  |  | Methionine | 43.5±8.0 |
|  |  | Proline | 109.4±7.6 |
|  |  | Taurine | 115.7±12.9 |
|  |  | Valine | 114.7±12.5 |

**Supplementary Data Table 5**. Matrix effect observed at two concentrations (C3= mid-range of the calibration curve and C5=upper limit of the calibration range).

| Compounds | Matrix effect ± SD (%) at concentration level C3 | Matrix effect ± SD (%) at concentration level C5 |
| --- | --- | --- |
| ORGANIC ACIDS |  |  |
| 2-Hydroxybutanoic acid | 82.0±9.1 | 82.3±2.1 |
| Lactic acid | 86.3±3.5 | 87.5±1.8 |
| Malic acid | 68.6±11.2 | 66.7±0.3 |
| 2-Oxoglutaric acid | 90.3±13.9 | 80.0±1.4 |
|  |  |  |
| AMINO ACIDS |  |  |
| Asparagine | 116.8±6.4 | 105.8±0.5 |
| Choline | 79.5±3.4 | 82.5±1.2 |
| Creatine | 99.9±1.7 | 99.6±1.2 |
| Creatinine | 57.8±0.8 | 65.7±1.3 |
| Leucine | 97.3±2.9 | 98.1±1.5 |
| Methionine | 30.6±2.1 | 35.3±0.6 |
| Proline | 60.8±2.0 | 66.9±1.0 |
| Taurine | 81.9±2.1 | 90.0±1.2 |
| Valine | 43.9±1.7 | 51.2±1.2 |


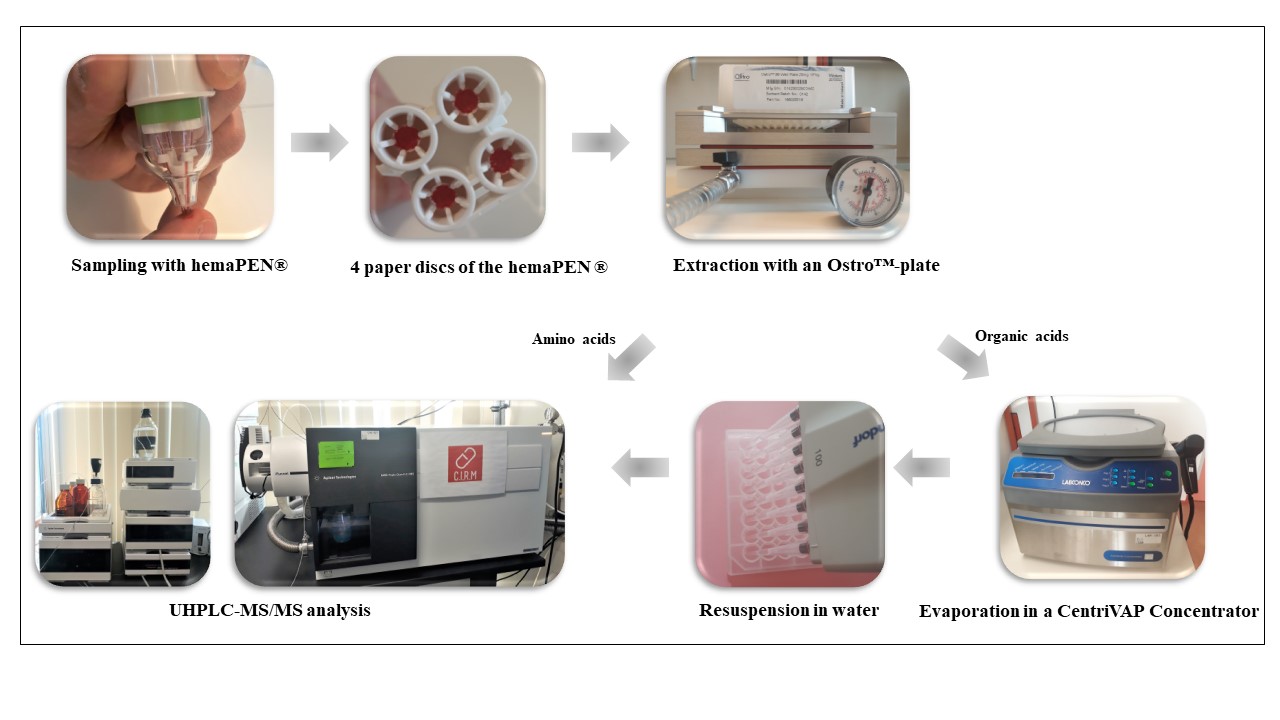


**Supplementary Data Figure 1**. *Sample collection and sample preparation method.*

Regression model: Weighted (1/x) quadratic regression. Concentration range: 640-6400 µM

R²: 0.9947 (series 1); 0.9838 (series 2) 0.9980 (serie s3)

Regression model: Weighted (1/x²) quadratic regression. Concentration range: 15-150 µM

R²: 0.9965 (series 1); 0.9771 (series 2) 0.9798 (series 3)

Lactic acid

2-Hydroxybutanoic acid

Regression model: Weighted (1/x) linear regression. Concentration range: 2.4-24 µM

R²: 0.9815 (series 1); 0.9925 (series 2) 0.9813 (series 3)

2-Oxoglutaric acid

Malic acid

Regression model: Weighted (1/x²) quadratic regression. Concentration range: 6-60 µM

R²: 0.9902 (seriess 1); 0.9679 (serie 2) 0.9786 (series 3)

Regression model: Weighted (1/x) quadratic regression. Concentration range: 5.5-55 µM

R²: 0.9913 (series 1); 0.9796 (series 2) 0.9932 (series 3)

Regression model: Weighted (1/x) quadratic regression. Concentration range: 14-140 µM

R²: 0.9921 (series 1); 0.9826 (series 2) 0.9833 (series 3)

Choline

Asparagine

Creatinine

Creatine

Regression model: Weighted (1/x) quadratic regression. Concentration range: 27-270 µM

R²: 0.9958 (series 1); 0.9939 (series 2) 0.9947 (series 3)

Regression model: Weighted (1/x) quadratic regression. Concentration range: 92.5-925 µM

R²: 0.9893 (series 1); 0.9893 (series 2) 0.9883 (series 3)

Regression model: Weighted (1/x) quadratic regression. Concentration range: 5.5-55 µM

R²: 0.9952 (series 1); 0.9934 (series 2) 0.9916 (series 3)

Regression model: Weighted (1/x²) quadratic regression. Concentration range: 28-280 µM

R²: 0.9926 (series 1); 0.9789 (series 2) 0.9803 (series 3)

Methionine

Leucine

Regression model: Quadratic regression. Concentration range: 41.5-415 µM

R²: 0.9978 (series 1); 0.9961 (series 2) 0.9953 (series 3)

Regression model: Weighted (1/x) quadratic regression. Concentration range: 55-550 µM

R²: 0.9984 (series 1); 0.9963 (series 2) 0.9944 (series 3)

Taurine

Proline

Valine

Regression model: Weighted (1/x) quadratic regression. Concentration range: 56-560 µM

R²: 0.9898 (series 1); 0.9921 (series 2) 0.9746 (series 3)

**Supplementary Data Figure 2**. Accuracy profiles obtained with the most appropriate regression models. Plain red line represents the relative bias, dashed blue lines represent the β-expectation limits and dashed black lines represent the acceptance limits. Dots represent the relative error of the back calculated concentrations and are plotted with respect to their targeted concentration. Blue dots=series 1 of pre-validation; red dots= series 2 of pre-validation and green dots= series 3 of pre-validation. R²=coefficient of determination.

**Supplementary Data Figure 3.** Carry-over (mean±SD) observed with these methods. n=3

**Supplementary Data Figure 4.** Levey-Jennings chart obtained with QC samples for 2-OH butanoic acid. Blue line represents the mean, green dotted lines represent mean±1SD, red dotted lines represent mean±2SD and grey dotted lines represent mean±3SD.
